# Normal transferrin glycosylation does not rule out severe ALG1 deficiency

**DOI:** 10.1002/jmd2.12415

**Published:** 2024-04-16

**Authors:** Inez Bosnyak, Mustafa Sadek, Wasantha Ranatunga, Tamas Kozicz, Eva Morava

**Affiliations:** ^1^ Department of Clinical Genomics Mayo Clinic Rochester Minnesota USA; ^2^ Department of Anatomy University of Pécs, Medical School Pécs Hungary; ^3^ Department of Laboratory Medicine and Pathology Mayo Clinic Rochester Minnesota USA; ^4^ Department of Biophysics University of Pécs, Medical School Pécs Hungary

**Keywords:** ALG1, CDG, congenital disorders of glycosylation, transferrin

## Abstract

ALG1‐CDG is a rare, clinically variable metabolic disease, caused by the defect of adding the first mannose (Man) to N‐acetylglucosamine (GlcNAc_2_)‐pyrophosphate (PP)‐dolichol to the growing oligosaccharide chain, resulting in impaired N‐glycosylation of proteins. N‐glycosylation has a key role in functionality, stability, and half‐life of most proteins. Therefore, congenital defects of glycosylation typically are multisystem disorders. Here we report a 3‐year‐old patient with severe neurological, cardiovascular, respiratory, musculoskeletal and gastrointestinal symptoms. ALG1‐CDG was suggested based on exome sequencing and Western blot analysis. Despite her severe clinical manifestations and genetic diagnosis, serum transferrin glycoform analysis was normal. Western blot analysis of highly glycosylated proteins in fibroblasts revealed decreased intercellular adhesion molecule 1 (ICAM1), but normal lysosomal associated membrane protein 1 and 2 (LAMP1 and LAMP2) expression levels. Glycoproteomics in fibroblasts showed the presence of the abnormal tetrasacharide. Reviewing the literature, we found 86 reported ALG1‐CDG patients, but only one with normal transferrin analysis. Based on our results we would like to highlight the importance of multiple approaches in diagnosing ALG1‐CDG, as normal serum transferrin glycosylation or other biomarkers with normal expression levels can occur.

**Synopsis**

Normal transferrin glycosylation results do not exclude the diagnosis of ALG1‐CDG even in young individuals with severe multisystem involvement.

## 1 INTRODUCTION

Congenital disorders of glycosylation (CDG) are rare genetic disorders caused by impaired glycoprotein and glycolipid glycan synthesis and attachment.^1^, ^2^ More than 160 CDG are known, the largest subset are N‐linked glycosylation defects.^3^, ^4^ N‐linked glycosylation plays a fundamental role in several biological processes, such as protein folding,^5^ endoplasmic reticulum (ER) homeostasis,^6^ neuronal function,^7^ and placentation.^8^

Synthesis of N‐glycans is a complex, multistep mechanism that takes place in the cytosol, ER and Golgi apparatus.^9^, ^10^ Nucleotide activated sugar molecules are transferred to dolichol located in the ER membrane. After phase I, two N‐acetylglucosamine (GlcNAc) and five mannose (Man) residues are attached to the cytoplasmic side of dolichol.^3^, ^11^, ^12^, ^13^

N‐linked glycosylation errors can occur in lipid‐linked oligosaccharide assembly, transfer of glycans to protein or during processing.^1^ CDG‐I are caused by defective dolichol‐linked oligosaccharide complex synthesis or transfer (cytosol and ER), while impaired processing in the ER and Golgi apparatus leads to CDG‐II.^14^

**FIGURE 1:**
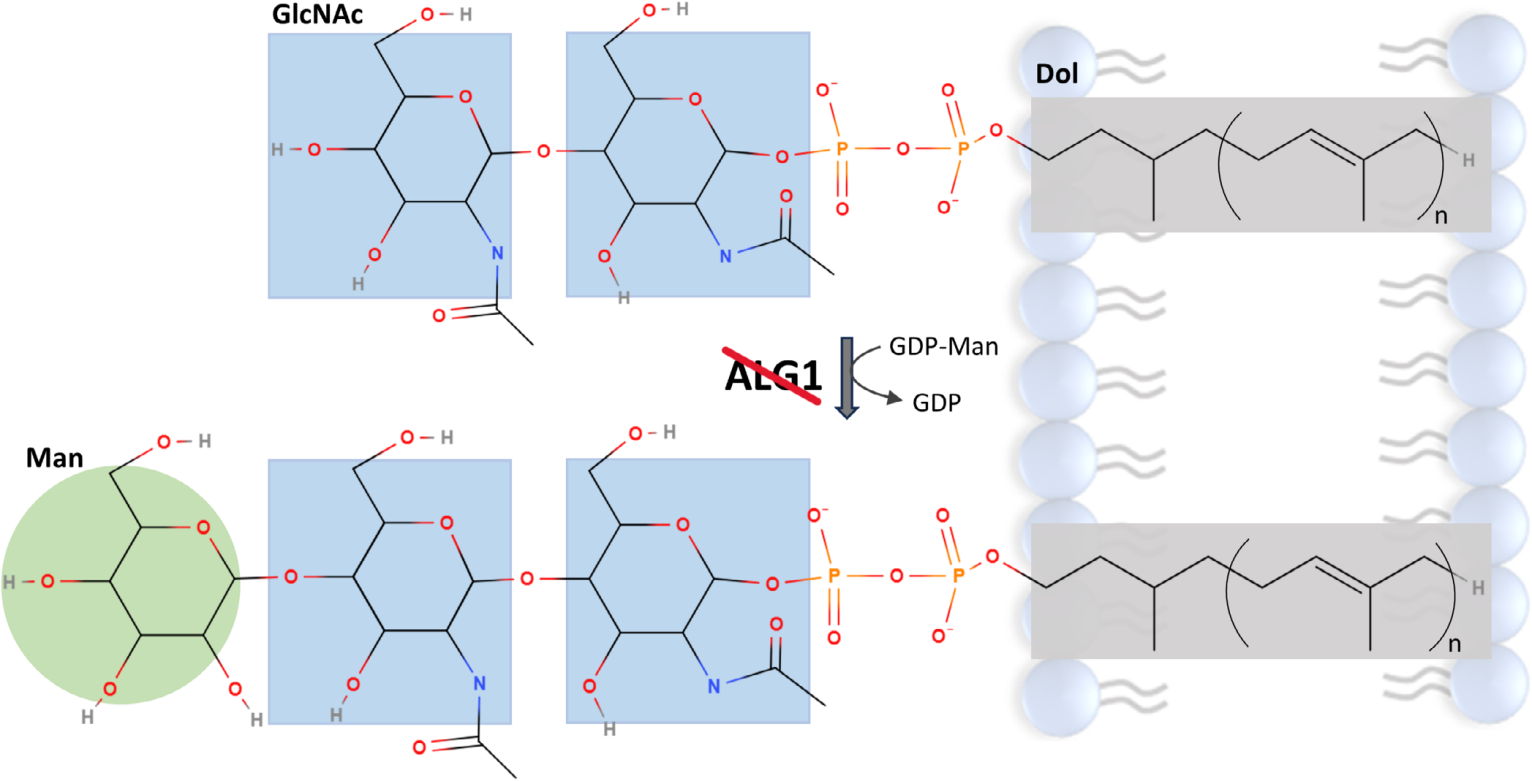
Schematic drawing of impaired ALG1 function. Dol, dolichol; GlcNAc, N‐acetylglucosamine; Man, mannose.

The asparagine‐linked glycosylation 1 (ALG1) protein belongs to a group of enzymes that catalyze the first steps of the oligosaccharide synthesis.^15^ It is responsible for adding the first Man to GlcNAc_2_‐PP‐dolichol.^16^ Its defect generates thus a CDG‐I (Figure 1). ALG1‐CDG (Online Mendelian Inheritance in Man [OMIM] #608540) is one of the most common CDG.^17^ Since its discovery in 2004^18^, ^19^, ^20^ more than 40 gene variants have been described.^21^

Most patients suffer from neurological involvement: hypotonia, intellectual disability, seizures and microcephaly. There is often cerebral/cerebellar atrophy. Besides these, ocular manifestations (especially strabismus and nystagmus), hematological (coagulation abnormalities) and skeletal defects (scoliosis, kyphosis, contractures), gastrointestinal problems (most commonly chronic diarrhea), and dysmorphic facial features are often present.^22^, ^23^ Liver and kidney abnormalities may also occur.^16^, ^17^, ^22^, ^24^ Most ALG1‐CDG phenotypes are also seen in other CDG, such as in the most common phosphomannomutase 2 (PMM2)‐CDG.^22^

The severity of the disease presentation varies over a wide spectrum. In some cases, just mild symptoms develop but early death can also occur.^16^, ^21^ According to recent data, congenital nephrotic syndrome, agammaglobulinemia and hydrops are associated with a severe outcome.^17^

### 1.1 Patient report

A 3‐year‐old girl was referred to our clinic with a suspected genetic diagnosis of ALG1‐CDG. Consanguinity was not reported in her family history. The maternal ancestry of the patient was Native American and Caucasian and her paternal ancestry is German. She was born at 26‐week gestation by emergency cesarean section due to a complete maternal placental abruption. Appearance, pulse, grimace, activity and respiration (Apgar) score was 6 at 1 min and 8 at 5 min. The baby needed intubation for 3 weeks, then required continuous positive airway pressure (CPAP) and high flow oxygen, which caused significant facial swelling. During her hospitalization, she barely moved and rarely seemed awake. She did not try to initiate a suction, so a gastrostomy tube was placed. Left sensorineural hearing loss was detected on infant hearing test. The metabolic newborn screening was normal.

At the age of 0.6 year she developed epileptic spasms. Routine electrocardiogram (ECG) initially showed burst suppression and multifocal spikes. Head ultrasound showed ventriculomegaly without intraventricular hemorrhage. Brain magnetic resonance imaging (MRI) showed extensive white matter volume loss, optic nerve atrophy, hypoplastic brainstem, and cerebellar vermis.

Her past medical history included cardiovascular (ventricular septum defect, Wolff–Parkinson–White syndrome), and respiratory symptoms (acute respiratory distress syndrome, hydrothorax, chronic respiratory failure, obstructive sleep apnea, restrictive lung disease, ineffective airway clearance). She was hospitalized at age 2 due to respiratory failure requiring intubation and ventilatory support. In addition, she had gastrointestinal symptoms (constipation, gastroesophageal reflux disease), a circadian rhythm sleeping disorder and muscle hypotonia.

At the age of 1 year she developed hypsarrhythmia, epileptic spasms with a striking for the degree of discontinuity and tonic seizures. Later on, the latter occurred daily. At the age of 3 years she showed multiple subtle epileptic spasms and a burst suppression pattern during sleep as well as focal seizures. In addition, at the age of 3 years she developed focal seizures and myoclonic seizures. Her overall course is consistent with Ohtahara syndrome. Her current therapy includes cannabidiol, clonazepam, zonisinamide, and levetiracetam.

At age 3 she cannot smile, does not have head control, cannot sit, and does not crawl. Her vocalization consists of crying and happy sounds. She has repetitive oral movements. Fine motor skills are also delayed. She can briefly grip an object. According to parents she is awake only a few hours a day, more at night. She rarely moves spontaneously, and her condition has worsened since discharge from the NICU. She receives physical‐, occupational‐, vision‐, and speech therapy.

Physical examination showed a drowsy general appearance, microcephaly, bifrontal narrowing, highly arched eyebrows, widely spaced eyes, short nose, high palate, low set ears, strabismus, no tracking eye movements, finger contractures in both hands, arachnodactyly, and short toes.

The patient's clinical status was evaluated based on Nijmegen Pediatric CDG Rating Scale (NPCRS),^25^ which scoring system includes the current function (score: 17), the system specific involvement (score: 11) and the current clinical assessment (score: 13). Her overall score was 41 out of 100, which quantifies the described severe phenotype. (Table S1A).

Whole exome sequencing showed a maternally inherited known pathogenic variant in *ALG1* (c.1187+3A>G) and a paternally inherited variant in *ALG1* of unknown significance (c.544G>A/ p. Glu182Lys).^26^

Interestingly, her carbohydrate deficient transferrin panel was normal: mono‐oligo/di‐oligo (0.02 Control: <0.06), a‐oligo/di‐oligo (0.003 Control <0.011) ratios were without abnormalities. (Table S2 and Figure S1A) Laboratory results showed slightly elevated antithrombin activity and slightly decreased total protein level, otherwise the results were within thresholds. Urine tetrasacharide analysis was normal. Tetrasacharide analysis by glycoproteomics in fibroblasts however showed the increased presence of the biomarker tetrasaccharide (NeuAc‐Gal‐GlcNAc2) from a variety of proteins.^27^

## 2 METHODS

We evaluated the patient carrying biallelic variants in *ALG1* who was enrolled and followed by the Frontiers of Congenital Disorders of Glycosylation Consortium (FCDGC) natural history study (institutional review board [IRB]: 19‐005187; NCT04199000). Data were collected prospectively, and retrospective data were available as well, as the patient was previously followed‐up according to standard of care.

Clinical residual material (patient‐derived skin fibroblasts) for expression analysis (Western Blot) was used upon written informed consent from both parents of affected individuals under Mayo Clinic *IRB:16‐004682*. The study was performed according to the declaration of Helsinki and its later amendments and in accordance with the ethics committees at each institution.

### 2.1 Targeted tetrasaccharide analysis

The patient‐derived fibroblast and control were included in the in vitro glycoproteomics studies under IRB protocol (IRB: 19–005187). For Glycoproteomics MAX‐enriched samples were analyzed by parallel reaction monitoring for selected candidate glycopeptides. This method is more suitable for analysis in a clinical setting due to lower mass spectrometry analysis time as well as enhanced sensitivity. The same age‐matched control fibroblast was included in the analysis. Cell lysis, protein digestion and glycoprotein enrichment were performed as described previously.^27^ Previous studies had already identified a Xeno‐oligosaccharide (tetrasaccharide; NeuAc‐Gal‐GlcNAc2) in the serum and fibroblasts of type I CDG patients through released N‐glycan analysis.^28^, ^29^ Three glycopeptide candidates (LAMP1, CD44, and tenascin) were selected for targeted analysis based on previous tandem mass tag (TMT)‐based LC–MS/MS.^27^, ^28^

### 2.2 Cell culture

Fibroblasts cells from patient and healthy controls were cultured in Minimal Essential Medium (MEM media Thermofisher) at 37°C incubator until they reached to 90%–100% confluency and harvested by scraping in phosphate buffered saline (PBS). Cell pellets were stored in −80°C after spinning down at 2000 rpm for 10 min and removing supernatants.

### 2.3 Western blot

Harvested fibroblast cell pellets from the patient and controls were lysed in radio‐immunoprecipitation assay (RIPA) buffer supplemented with protease inhibitors (SIGMA, USA) and in 1 mM phenylmethanesulfonyl fluoride (PMSF). Protein concentrations were determined using Pierce bicinchoninic acid (BCA) assay (Thermo Fisher Scientific, USA).

Required amount of protein samples (15 μg protein/lane for ICAM1, 20 μg/lane for ALG1, 30 μg/lane for LAMP1 and LAMP2) were mixed with loading buffer (25% Laemmli buffer and 10% sample reducing agent (Cat# NP009 Invitrogen)). Samples were boiled for 30 min at 70°C (for ICAM1) or denatured for 5 min at 95°C (for LAMP1, LAMP2 or ALG1 in the presence of loading buffer containing 25% Laemmli buffer and 10% β‐mercaptoethanol), loaded into 10% NuPAGE Bis‐Tris gel (for ICAM1 and ALG1) or 4%–12% NuPAGE Bis‐Tris gels for LAMP1and LAMP2. Proteins were separated electrophoretically at 200 V for 1 h at room temperature (RT) and protein bands were transferred to nitrocellulose membrane at 35 V for 3 h at 4°C. The membrane was then blocked using blocking buffer (Fish Serum Blocking Buffer (Thermo Fisher Scientific, USA) for ICAM1 and LAMP2, bovine serum albumin (BSA)/(Tris buffered saline with 0.1% Tween 20) TBST for LAMP1 and ALG1) for an hour at 4°C for ICAM1 and LAMP2 or RT for LAMP1 and ALG1, followed by incubation with the primary antibodies at 4°C for indicated time (mouse monoclonal ICAM1 1:500 Cat# sc8439 (Santa Cruz) 24 h; mouse LAMP2 1:250 Cat# 66301‐1‐Ig (Proteintech) 72 h, rabbit anti‐LAMP1 1:500 Cat# ab24170 (abcam) 24 h; rabbit ALG1 1:500 Cat# 12872‐1‐AP (Thermo Fischer) 24 h; along with rabbit β‐actin at 1:10 000 Cat# AC026 ABclonal or Mouse β‐Actin Cat# AC004 ABclonal at 1:10 000). Afterwards PBST (phosphate buffered saline with 0.1% Tween 20) was used for washing the blot (5 min each, 6 times) at RT for ICAM1 and LAMP2 or TBST for LAMP1 and ALG1 (10 min each, 3 times), followed by the incubation with secondary antibodies (Donkey anti‐Mouse Dy‐light 800 Cat# SA510172 Invitrogen; Donkey anti‐Rabbit Dy‐light 680 Cat# SA5‐10042 Invitrogen) for 1 h at 4°C for ICAM1 and LAMP2 or TBST at RT for LAMP1 and ALG1. Blots were kept in either PBS (ICAM1 and LAMP2) or Tris buffered saline (TBS) (LAMP1 and ALG1) before visualizing the bands. Odyssey Fc system and Li‐Cor Odyssey Image Studio version 3.1 (Li‐Cor Biosciences, Lincoln, NE, USA) were used to visualize and quantify the protein expression levels.

Odyssey Fc system and Li‐Cor Odyssey Image Studio version 3.1 (Li‐Cor Biosciences, Lincoln, NE, USA) were used to visualize and quantify the proteins expression levels. Quantified data were analyzed using the Excel and GraphPad Prism software.

### 2.4 Literature review

We reviewed the clinical, metabolic and genetic characteristics of all reported patients with pathogenic variants in the ALG1 gene.

## 3 RESULTS

Western blot analysis showed almost no detectable ALG1 protein in the patient's fibroblasts, suggesting ALG1‐CDG diagnosis, upon exome sequencing results. Unfortunately no yeast complementation study was possible for this variant.

**FIGURE 2:**
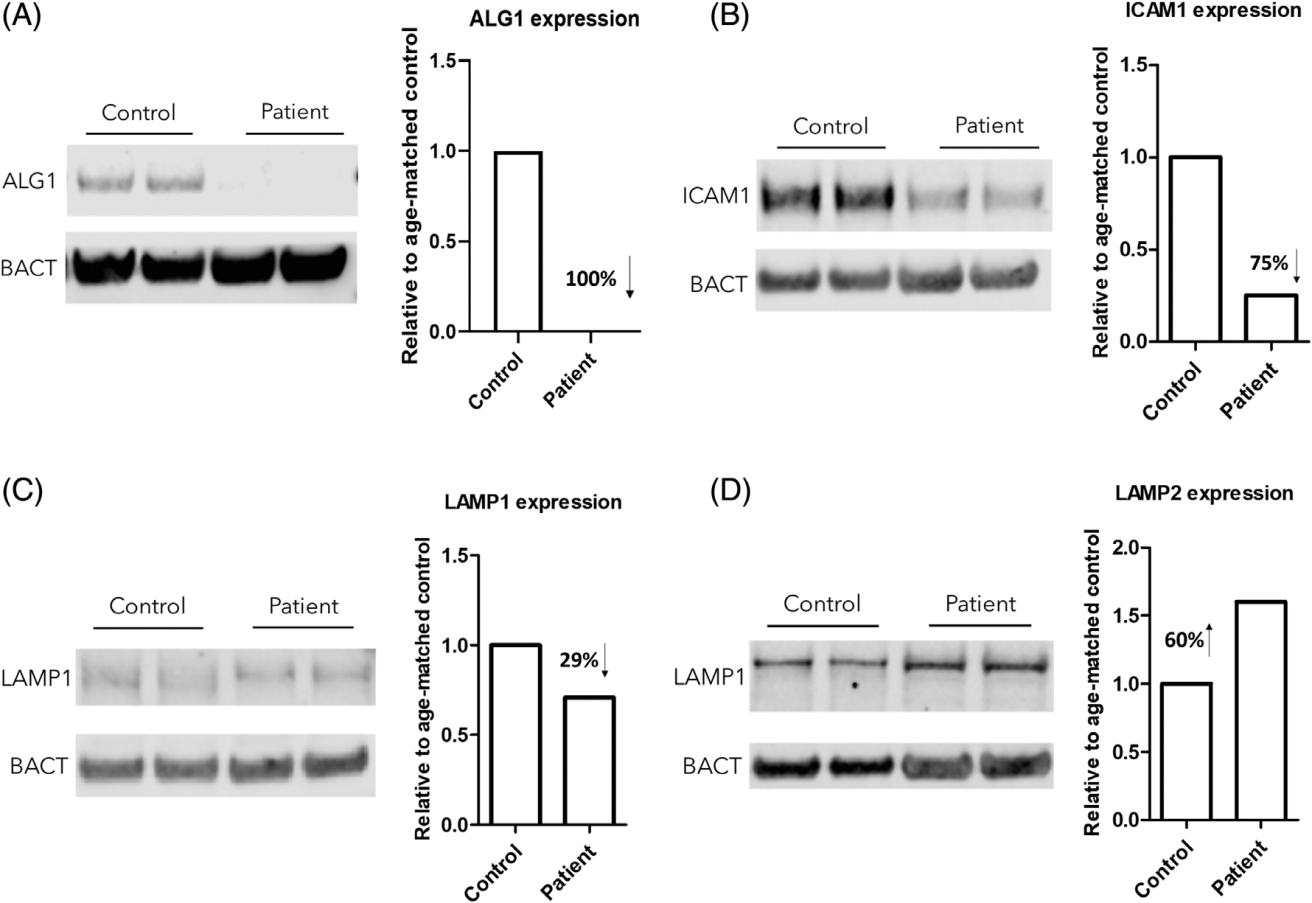
Western blot results. (A) expression of ALG1 enzyme, (B) expression level of ICAM1 protein, (C) expression level of LAMP1 protein, and (D) expression level of LAMP2 protein.

Mature ICAM1 abundance was 75% decreased in the patient's cells compared to age‐matched control. Interestingly, no relevant change was observed in abundance or migration of LAMP1 and LAMP2 proteins. (Figure 2).

By proteomics analysis there was a reduction of ALG1 protein level by approximately 70% in patient fibroblasts compared to controls. Three target glycopeptides from LAMP1, CD44 and tenascin bearing the xeno‐tetrasaccharide (NeuAc‐Hex‐HexNAc_2_; Figure S1B) were detected in fibroblasts from the affected individuals but not in control fibroblasts.

We found 86 patients diagnosed with ALG1‐CDG in the medical literature. All but one had abnormal transferrin glycoforms by different analytic methods.

Twenty‐two of the reported patients were certainly alive at 1 year of age, 17 children lived at 5 years of age, 9 among them survived at least to 10 years of age and 3 patients lived longer than 15 years. (Table 1) The oldest described person with ALG1‐CDG was 24 years old.^30^

**TABLE 1:** Transferrin glycosylation results and age of previously reported ALG1‐CDG cases.

Number of patients	Age	Tf analysis	Author	Date
1	Younger than 1 year	+	Grubenmann	2004
1	Died at 2 weeks of age	+	Schwarz	2004
2	Died at 10 months of age	+	Kranz	2004
	Died at 11 weeks of age			
5	4 months at diagnosis, died at 4 years and 9 months	+ (based on Western blot)	Dupré	2010
	16 months at diagnosis, alive at age 9 years			
	1 year and 6 months at diagnosis, alive at age 11 years			
	10 months at diagnosis, alive at age 10			
	3 years and 7 months at diagnosis, alive at age 14			
7	Alive at 21 years	+	Morava	2012
	Died at age 10 years			
	Alive at 5 years and 8 months of age			
	Alive at 10 years and 9 months of age			
	Alive at 6 years and 2 months of age			
	Died at 5 months			
	Alive at 10 years and 1 month of age			
1	Died at 44 days	NA	Snow	2012
1	Died at 3 months and 23 days of age	+	Rohlfing	2014
2	4 months at diagnosis, alive at 10 months	+	Fiumara	2016
	NA	NA		
11	Died before 1 year of age	+	Ng	2016
6	Alive at 1 year of age			
22	NA			
1	Died at 1 year of age	+	Barba	2016
2	1 year old at diagnosis	+	Perez‐Cerda	2017
	8 years old at diagnosis			
2	1 year at diagnosis, alive at age of 10 years	+	Pereira	2017
	2 years at diagnosis, alive at 11 years			
2	Alive at 24 years of age	Probably type I Tf pattern	Medrano	2019
	Alive at 5 years of age			
1	Pregnancy was terminated at 24 weeks of gestational age	NA	Lei	2020
3	Last follow‐up 5 months old, patient died	− (1 patient) + (2 patients)	Bogdanska	2021
	Last follow‐up 5 months old, patient died			
	Last follow‐up 10 months old, no further data			
1	NA	NA	Fang	2021
1	15 years old	+	Gonzalez‐Dominiguez	2021
1	15 months old	+	Öncül	2021
4	NA	+	Abu Bakar	2022
1	Died at 1 year	Diagnosis based on genetics, NA	Yang	2022
7	At diagnosis: 0.5 years old, alive at 10.67 years	Probably type I	Dang Do	2023
	0.58 years old			
	NA			
	NA			
	0.83 years old at diagnosis, alive at 11.5 years			
	0.06 years old at diagnosis, now 0.33‐year‐old			
	4 years old			
1	13 months	Diagnosis based on genetics, NA	Xue	2023

*Note*: + is Type I Tf pattern; − is normal Tf panel; NA is no data.

## 4 DISCUSSION

ALG1‐CDG (OMIM #608540) is a rare metabolic disorder, which is caused by homozygous or compound heterozygous disease‐causing variants on chromosome 16p13 in the gene encoding beta‐1,4‐mannosyltransferase. The diagnosis is usually based on genetic testing, after finding a type 1 serum transferrin isoelectrofocusing pattern.^31^ Although the serum transferrin profile could be normal in the first weeks of life in CDG patients^32^ or CDG patients with Golgi disruption^33^ this has been reported abnormal in ALG1‐CDG patients in early childhood. However, the possibility of spontaneous improvement in transferrin glycosylation in young PMM2‐CDG patients is known.^34^

Based on our literature review, 86 patients have been described since the discovery of the disease and only one of them showed a normal transferrin glycosylation. (Table 1). Chromosome 16p13.3 deletion involving ALG1 gene was described in case of the 10‐month‐old boy. Like our patient, the boy has severe clinical manifestations with muscle hypotonia, seizures, microcephaly, failure to thrive, among others. As far as we know, our patient is the second very severe ALG1‐CDG patient without transferrin abnormalities.^35^

Western blot results from collected fibroblasts showed the absence of ALG1 protein in our patient. ICAM1, LAMP1, and LAMP2 were also analyzed. They are highly glycosylated proteins, so their decreased expression (due to decreased glycosylation) could support our diagnosis.^36^ ICAM1 was decreased but not LAMP1 and LAMP2. Altered expression levels of these marker proteins however could be due to either glycosylation changes or change in gene expression instead of glycosylation defect, making them difficult to interpret.^37^ In 2015, a new biomarker, a urinary tetrasaccharide, consisting of one sialic acid, one galactose, and two N‐acetylglucosamines (NeuAc‐Gal‐GlcNAc2) was discovered for ALG1‐CDG, which can support the diagnosis made by other methods. This biomarker can be measured in urine and blood and has so far been detected in several affected individuals.^22^, ^38^, ^39^

Here we report on a novel ALG1‐CDG patient with severe clinical manifestations, but normal transferrin glycosylation in blood, and normal urine tetrasaccharide, but increased protein‐tetrasaccharide in fibroblasts by glycoproteomic analysis. The presence of abnormal glycosylation in fibroblasts, the low abundance of ALG1 expression and the presence of the abnormal tetrasaccharide specific for early N‐glycosylation defects is highly suggestive for the pathogenicity of the described variant. We would like to draw attention to the importance of multiple approaches in diagnosing ALG1‐CDG, as a normal transferrin pattern does not exclude this diagnosis and additional screening markers (Western blot of glycosylated LAMP1 and LAMP2 in fibroblasts) can be normal too.

## AUTHOR CONTRIBUTIONS

IB, MS, and EM were involved in conceptualization and wrote the manuscript. WR and TK were involved in the data evaluation. All authors were involved in the interpretation of data and reviewing and editing the manuscript.

## FUNDING INFORMATION

Eva Morava, Tamas Kozicz, and Mustafa Sadek are supported by the grant titled Frontiers in Congenital Disorders of Glycosylation (1U54NS115198‐01) from the National Institute of Neurological Diseases and Stroke (NINDS) and the National Center for Advancing Translational Sciences (NCATS), National Institute of Child Health and Human Development and the Rare Disorders Clinical Research Network (RDCRN), at the National Insitute of Health Sciences.

## CONFLICT OF INTEREST STATEMENT

Inez Bosnyak, Mustafa Sadek, Wasantha Ranatunga, Tamas Kozicz, and Eva Morava have approved the manuscript and declare no conflict of interest.

## ETHICS STATEMENT

Patients included in this work are enrolled in the Frontier in CDG Consortium (FCDGC) natural history study (institutional review board (IRB) 19‐005187; https://clinicaltrials.gov/ct2/show/NCT04199000?cond=CDG&draw=2&rank=4). Written informed consent was obtained from the legally authorized representatives of the subjects prior to study initiation. All procedures followed were in accordance with the ethical standards of the responsible committee on human experimentation (institutional and national) and with the Helsinki Declaration of 1975, as revised in 2000. Written informed consent was obtained from the patient's parent for collection of samples and publication of medical data.

## Supporting information


**Figure S1.** (A) Mass spectrometry of transferrin in a patient with ALG1‐CDG showing a normal pattern. (B) Schematic figure of the protein linked xeno‐tetrasacharide.

**Table S1.** Laboratory results of the carbohydrate‐deficient transferrin panel and the ApoCIII glycoform values.

**Table S2.** Nijmegen Pediatric CDG Rating Scale (NPCRS): 2–11 years, results of our patient.

## Data Availability

This manuscript has no associated data or supporting data.
