# Supplementary figures and images for: Normal transferrin glycosylation does not rule out severe ALG1 deficiency
Source: JIMD Rep. 2024 Apr 16;65(3):135–43. doi: 10.1002/jmd2.12415 (PMC11078713; doi:10.1002/jmd2.12415)

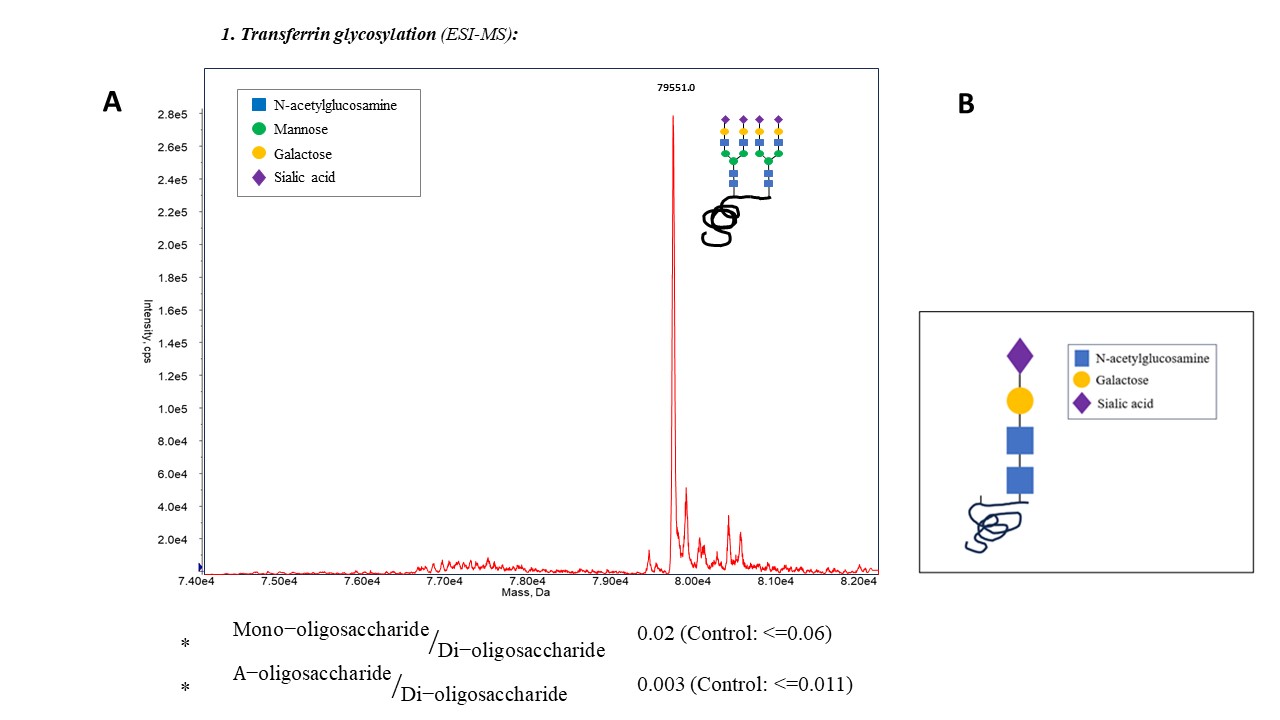

Supplement: Supplementary file 1 — Figure S1. (A) Mass spectrometry of transferrin in a patient with ALG1‐CDG showing a normal pattern. (B) Schematic figure of the protein linked xeno‐tetrasacharide. [file JMD2-65-135-s001.jpg]
