# Supplementary material for: Normal transferrin glycosylation does not rule out severe ALG1 deficiency
Source: JIMD Rep. 2024 Apr 16;65(3):135–43. doi: 10.1002/jmd2.12415 (PMC11078713; doi:10.1002/jmd2.12415)
Supplement: Supplementary file 2 — Table S1. Laboratory results of the carbohydrate‐deficient transferrin panel and the ApoCIII glycoform values. [file JMD2-65-135-s002.docx]

|  | Normal | Patient |
| --- | --- | --- |
| Mono-oligo/Di-oligo Ratio | <=0.06 | 0.02 |
| A-oligo/Di-oligo Ratio | <=0.011 | 0.003 |
| Tri-sialo/Di-oligo Ratio | <=0.05 | 0.01 |
| Apo CIII-1/Apo CIII-2 Ratio | <=2.91 | 2.54 |
| Apo CIII-0/Apo CIII-2 Ratio | <=0.48 | 0.11 |

*Suppl. Table 1.* Laboratory results of the carbohydrate-deficient transferrin panel and the ApoCIII glycoform values
