# Supplementary material for: Normal transferrin glycosylation does not rule out severe ALG1 deficiency
Source: JIMD Rep. 2024 Apr 16;65(3):135–43. doi: 10.1002/jmd2.12415 (PMC11078713; doi:10.1002/jmd2.12415)
Supplement: Supplementary file 3 — Table S2. Nijmegen Pediatric CDG Rating Scale (NPCRS): 2–11 years, results of our patient. [file JMD2-65-135-s003.docx]

| **Section 1: Current Function** | |
| --- | --- |
| Vision | 2. Moderate. Visual impairment not fully corrected with glasses or inattention to large objects in visual field |
| Hearing | 1. Mild. Requires regular repetition/raised voice or not reacting to loud sounds (e.g., clapping) left |
| Communication | 3. Severe. Not communicating effectively with parents (irrespective of methods) |
| Feeding | 3. Severe. Exclusive enteral feeding (gastrostomy/NG tube). Nil by mouth |
| Self-care | 3. Severe. Reliant on parents with no contribution to self care |
| Mobility | 3. Severe. Wheelchair/carrier dependent |
| Educational achievement | 2. Moderate. Attending special school/nursery |
| Section I score: **17** | |
| **Section II: System Specific Involvement** | |
| Seizures | 2. Moderate. >5 generalized tonic-clonic seizures/month or >20 absence or myoclonic seizures/month |
| Encephalopathy | 2. Moderate. Abnormal mood and behavior or excessive sleepiness |
| Bleeding diathesis or coagulation defects | 0. None |
| Gastrointestinal | 1. Mild. Mild constipation or unexplained vomiting/diarrhea <1/week |
| Endocrine | 0. Normal |
| Respiratory | 3. Severe. Abnormal respiration requiring artificial ventilation |
| Cardiovascular | 2. Moderate. Abnormal echocardiogram (e.g., cardiomegaly) or sustained/symptomatic arrhythmia on ECG or chronic pericardial fluid collection |
| Renal | 0. Normal |
| Liver | 0. Normal |
| Blood | 1. Mild. Anemia or thrombocytopenia only |
| Section II score: **11** | |
| **Section III: Current Clinical Assessment** | |
| Growth (height and weight) over preceding 6 months | 0. Normal. Following normal growth trajectory |
| Development over preceding 6 months | 5. No regression, global developmental delay with developmental progress |
| Vision with usual glasses. Acuity is based on vision in the better eye | 2. Moderate. Acuity worse than 6/18 but better than or equal to 6/60 or impaired fixation on large, brightly colored objects |
| Strabismus and eye movement | 1. Mild. Intermittent strabismus or ptosis or impaired eye movement at extremities |
| Myopathy | 2. Moderate. Moderate symmetrical weakness (proximal>distal) limiting mobility |
| Ataxia | 0. Normal |
| Pyramidal | 3. Severe. Unable to make steps with aids primarily due to spasticity |
| Extrapyramidal | 0. Normal |
| Neuropathy | 0. Normal |
| Section III score: **13** | |

*Suppl. Table 2.* Nijmegen Pediatric CDG Rating Scale (NPCRS): 2-11 years, results of our patient
